# Biomimetic nanoplatform M-MPDA@CeO2 coated with macrophage membrane in acute lung injury: characterization and function evaluation

**DOI:** 10.1186/s12967-026-08380-y

**Published:** 2026-06-03

**Authors:** Yi Luo, Yanping Wei, Fu Wei, Lianshan Guo, Guoliang Xie, Lei Zhuoyi, Xiaomei Tang, Huihe Chen, Jianfeng Zhang, Zhengzhao Li

**Affiliations:** 1 Department of Emergency, The Second Affiliated Hospital of Guangxi Medical University, Nanning, Guangxi China; 2 Department of Intensive Care Unit, Wuming Hospital of Guangxi Medical University, Nanning, Guangxi 530119 China; 3 Department of Emergency, The Frist Peoples Hospital of Foshan, Foshan, Guangdong China; 4 Department of Emergency, Wuming Hospital of Guangxi Medical University, Nanning, Guangxi China; 5 The Emergency Department of Liuzhou Traditional Chinese Medicine Hospital, Guangxi, 545001 China

**Keywords:** M-MPDA@CeO_2_, Macrophage membrane, Acute lung injury, Post-translational modification, Nanomedicine

## Abstract

The pathogenesis of acute lung injury (ALI) is characterized by a dysregulated hyperinflammatory cascade initiated by activated pulmonary macrophages. Conventional nanoparticles exhibit limited therapeutic efficacy in ALI due to their insufficient penetration across the pulmonary mucus barrier and inability to inhibit the exacerbated inflammatory cascade within the pathogenic microenvironment. To address these limitations, this study developed a novel macrophage membrane-biomimetic nano-delivery system designed to enhance biocompatibility, achieve targeted delivery to inflammatory sites, and exert synergistic antioxidant effects. The nanocomposite was constructed by loading cerium dioxide (CeO_2_) nanoparticles onto mesoporous polydopamine (MPDA) and further functionalizing the surface with macrophage membranes through engineering strategies, yielding the M-MPDA@CeO_2_ formulation. The physicochemical properties of M-MPDA@CeO_2_ was systematically characterized, and its function in reactive oxygen species (ROS) scavenging, antioxidation, macrophage polarization was evaluated in vitro. Using an LPS-induced ALI mouse model, the therapeutic potential of M-MPDA@CeO_2_ was assessed in terms of anti-inflammatory and oxidative activities. Results demonstrated that the MPDA@CeO_2_ nanocomposite effectively scavenges ROS, alleviates LPS-induced oxidative damage, promotes M2 macrophage polarization, and selectively accumulates at inflammatory sites in ALI. This biomimetic nanoparticle drug delivery system offers a promising strategy for improving ALI treatment, with potential implications for both preclinical research and clinical translation.

**Supplementary Information:**

The online version contains supplementary material available at 10.1186/s12967-026-08380-y.

## Graphical Abstract

**Figure:**
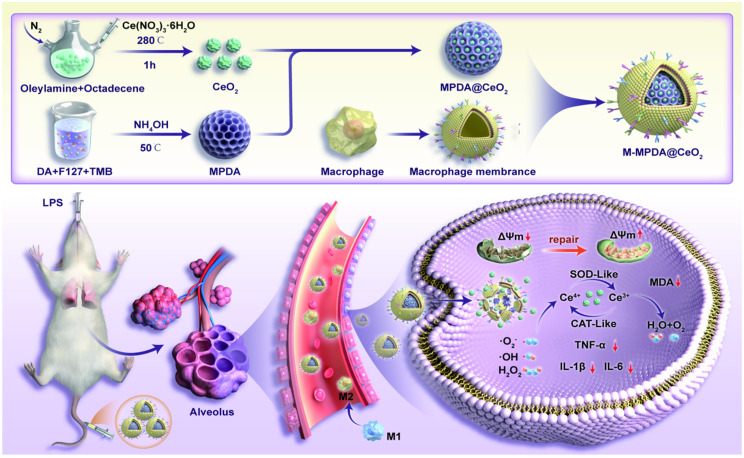

## Introduction

Acute Lung Injury (ALI) is a life-threatening and prevalent pulmonary inflammatory disorder pathologically featured with disruption of the alveolar-capillary membrane, resulting in excessive fluid and protein exudation between alveoli and capillaries [1, 2]. ALI, and its more severe manifestation, acute respiratory distress syndrome (ARDS), pose significant global public health challenges across high-risk populations of all age groups throughout the life cycle [3]. Despite progress in critical care, ARDS mortality rates remain troublingly high, and survivors often suffer from lasting physical and cognitive impairments [4]. The etiologies of ALI are diverse and complex, including direct factor such as toxic inhalation, pulmonary infections, and thoracic trauma, as well as indirect systemic triggers like severe sepsis, shock, and systemic inflammatory response syndrome [5]. Acute-phase inflammation worsens lung injury through neutrophil infiltration and pro-inflammatory mediator release, causing further damage to pulmonary endothelial and epithelial cells and perpetuating barrier breakdown [6]. Besides, excessive reactive oxygen species (ROS) significantly contribute by oxidizing cellular membranes, proteins, and nucleic acids, thereby amplifying tissue damage and inflammatory responses, worsening pulmonary dysfunction [7]. The sodium-potassium-chloride co-transporter isoform 1, which drives water transport via back transport of Na^+^ and Cl^-^ to the alveolar air space, is crucial in regulating alveolar fluid, and the concentration of potassium ions is necessary for monitoring intracellular dynamics in lung injury [8, 9]. These processes establish a vicious cycle of “inflammation-oxidative stress,” ultimately triggering alveolar epithelial cell apoptosis, pulmonary edema, and progressive respiratory failure.

The clinical management of ALI and ARDS remains a significant challenge. Current clinical therapeutic approaches are significantly constrained by the limitations of conventional drug delivery systems, which are plagued by issues such as poor drug solubility, uneven biodistribution, rapid metabolic clearance, lack of targeting specificity, low in vivo stability, suboptimal bioavailability, and considerable adverse effects [10]. Even when employing natural compounds with inherent anti-inflammatory and antioxidant properties, these systems often fail to achieve effective therapeutic concentrations at pathological sites or realize their full pharmacological potential [11]. This critical situation necessitates the urgent development of novel drug delivery platforms capable of establishing stable targeted drug delivery systems to prolong therapeutic duration and enhance treatment efficacy, thereby overcoming the current clinical limitations in managing inflammatory diseases.

Nanotechnology-based drug delivery systems (NDDS) represent a transformative approach in modern medicine, designed to enhance drug precision and efficacy while minimizing toxicity. Within the precision medicine paradigm, targeted drug delivery systems (TDDS) have become a research frontier, specifically engineered to recognize and bind to particular cell populations through specialized ligands [12]. This targeted approach proves particularly valuable for treating conditions like ALI, where nanoparticle-based systems can concentrate medications at disease sites while sparing healthy tissues, thereby optimizing drug distribution and therapeutic outcomes. The application of nanoparticles (NPs) in ALI treatment demonstrates considerable promise, including the immune-liposome system [13] and the fluorinated peptide nanostructure [14]. While moderate ROS levels serve protective functions, their dysregulation disrupts oxidative balance, contributing to tissue damage through mechanisms like extracellular matrix degradation and alveolar cell apoptosis. Cerium dioxide nanoparticles (CeO_2_NPs) offer a sophisticated solution to ROS dysregulation through their dual enzyme-mimetic capabilities, simultaneously mimicking superoxide dismutase (SOD) and catalase (CAT) activities [15]. Their unique mixed-valence surface (Ce^3+^/Ce^4+^) enables both ROS neutralization and oxygen generation, though their full potential in pulmonary protection remains underexplored [16, 17]. Complementing these approaches, polydopamine (PDA)-based systems combine efficient drug loading through π-π stacking interactions with inherent antioxidant properties [18]. Recent innovations like mesoporous PDA (MPDA) nanoparticles and biomimetic cell membrane-coated NPs further enhance therapeutic potential. Macrophage membrane-coated NPs have gained significant attention for their excellent biocompatibility, immune evasion, and lesion-targeting capabilities [19]. By leveraging the natural inflammation-directed chemotactic ability of macrophages, macrophage membrane-coated NPs are able to actively accumulate at sites of inflammation or diseased tissue, significantly improving circulation time and targeting accuracy [20]. Together, these nanotechnology platforms demonstrate how precise engineering at the molecular level can revolutionize drug delivery and therapeutic outcomes.

This study reports the design and characterization of a novel nanocomposite material. This nanocomposite integrates ultrasmall cerium dioxide (CeO_2_) nanoparticles loaded onto mesoporous polydopamine (MPDA) and further functionalized with macrophage membranes via surface engineering, resulting in the M-MPDA@CeO_2_ construct (Graphical abstract). Furthermore, we further characterized the M-MPDA@CeO_2_, and evaluated its performance in ROS elimination, antioxidant, macrophage polarization, as well as its biocompatibility in vitro and in vivo. In ALI mouse, we assessed the therapeutic efficacy of M-MPDA@CeO_2_. By introducing a novel nanotechnology-based drug delivery system, this study aims to provide a high efficiency therapeutic paradigm for anti-inflammatory and anti-oxidative interventions in ALI.

## Materials and methods

### Synthesis of M-MPDA@CeO₂

#### Synthesis of CeO₂

A mixture of 500 mg Ce(NO₃)₃·6 H₂O and 200 mg trioctylphosphine oxide (TOPO) was placed in a 50 mL beaker. After adding 20 mL of anhydrous ethanol and 2 mL of deionized water, the solution was heated and stirred until the solutes dissolved completely. This solution was then transferred to a 20 mL glass flask, where 5 mL of octadecene (ODE) and 100 µL of oleylamine (OAm) were added. Under a nitrogen atmosphere, the mixture was heated to 280 °C for 1 h, forming a pale-yellow colloidal solution. After cooling to room temperature, the product was washed three times with anhydrous ethanol by centrifugation, discarding the supernatant each time. The precipitate was then dispersed in acetone for solvent exchange, centrifuged, and left open for 2 h to evaporate residual acetone. Finally, the product was lyophilized under vacuum, yielding a pale-yellow powder stored at -20 °C.

#### Synthesis of MPDA

150 mg of dopamine hydrochloride and 100 mg of Pluronic F127 were dissolved in 10 mL of deionized water and ethanol, respectively. The solutions were mixed, followed by the addition of 160 µL of 1,3,5-trimethylbenzene. The mixture was ultrasonicated in a water bath for 6 min. Subsequently, 375 µL of ammonia solution was added gradually while stirring magnetically, and the reaction proceeded at 50 °C for 2 h. The resulting product was centrifuged at 900 rpm for 10 min and washed thrice with ethanol and ultrapure water. The product was then lyophilized and stored at -20 °C.

#### Synthesis of MPDA@CeO₂

A 20 mg sample of MPDA powder was dispersed in 20 mL of anhydrous ethanol with stirring. Then, 5 mg of CeO₂ was introduced, and the mixture was magnetically stirred at room temperature for 12 h. The composite was collected by centrifugation at 900 rpm for 10 min, washed three times with anhydrous ethanol, and lyophilized to produce the MPDA@CeO₂ powder.

#### Extraction of macrophage membranes

##### Cell harvesting

RAW 264.7 murine macrophage cells were cultured in DMEM complete medium with 10% fetal bovine serum at 37 °C and 5% CO₂. Upon reaching 90% confluence, the cells were washed three times with phosphate-buffered saline (PBS). They were then scraped, transferred to a 1.5 mL centrifuge tube at a density of about 2 × 10⁷ cells/mL, and centrifuged at 100 rpm for 5 min at 4 °C. The supernatant was discarded.

##### Membrane isolation

The cell pellet was resuspended in membrane protein extraction buffer and incubated on ice for 15 min. The suspension was then homogenized 30 times at 4 °C using a pre-chilled homogenizer to ensure complete lysis. The homogenate was centrifuged at 200 rpm for 10 min at 4 °C. The supernatant was further centrifuged at 14,000 rpm for 30 min at 4 °C, yielding a pellet of macrophage membrane fragments. These fragments were resuspended in PBS and extruded 10–15 times through a 400 nm polycarbonate membrane using a liposome extruder to produce macrophage membrane-derived nanovesicles (MM NVs), which were stored at 4 °C for later use.

#### Synthesis of M-MPDA@CeO₂ nanocomposites

Macrophage membranes were applied to MPDA@CeO₂ nanoparticles using a co-extrusion technique. The extracted MM NVs were combined with MPDA@CeO₂ core nanoparticles in water at a 10:1 mass ratio. This mixture underwent 5 min of sonication with an ultrasonic disperser (30 kHz, 100 W) and was extruded 15 times through a 200 nm polycarbonate membrane via a liposome extruder. The resulting M-MPDA@CeO₂ nanocomposites were collected, vacuum-lyophilized, and stored at 4 °C.

#### Synthesis of fluorescently labeled nanocomposites

Fluorescently-labeled nanocomposites were synthesized by replacing the core of M-MPDA@CeO₂ with MPDA@Cy5.5. The Cy5.5 dye (excitation = 683 nm, emission = 703 nm) was incorporated into the nanocomposite structure. These fluorescent nanocomposites were employed for in vitro and in vivo fluorescence-based assessments.

#### Western blot validation of surface-specific proteins on M-MPDA@CeO₂

The retention of macrophage membrane proteins on the surface of M-MPDA@CeO₂ nanoparticles was confirmed through Western blotting (WB) analysis. Key proteins, including Integrin β1, CD47, and TNFα-2, were detected in raw macrophages, macrophage membrane-derived nanovesicles (MM NVs), and M-MPDA@CeO₂ samples. The presence of these characteristic macrophage membrane proteins on the surface of M-MPDA@CeO₂ nanoparticles demonstrated the successful integration of macrophage membranes onto the MPDA@CeO₂ nanoparticle platform.

### Material characterization

#### Transmission electron microscopy (TEM)

Transmission Electron Microscopy (TEM) is a high-resolution technique employing an electron beam to obtain detailed microstructural and morphological data. Unlike optical microscopy, TEM provides superior spatial resolution, allowing visualization of crystal structures and nanoscale features. In TEM, an electron beam penetrates the sample, and the transmitted electrons are focused to produce a high-resolution two-dimensional image based on scattering patterns. This study analyzed the morphologies of CeO₂ and M-MPDA@CeO₂ using a JEM-120EX tungsten filament TEM at an accelerating voltage of 120 kV. Samples were prepared by depositing dispersed nanoparticles onto ultrathin carbon-coated copper grids and drying them at room temperature before imaging.

#### Scanning electron microscopy (SEM)

Scanning electron microscopy (SEM) is a widely employed technique for high-resolution surface imaging at the nanoscale. This method utilizes a focused, high-energy electron beam generated by an electron gun to scan the sample surface. The interactions between the electron beam and the sample surface produce secondary and backscattered electrons, which are detected to generate detailed topographical images. In this study, the morphologies of MPDA and MPDA@CeO₂ were characterized using a Sigma300 SEM operated at an accelerating voltage of 5 kV. Samples were prepared by depositing aqueous nanoparticle suspensions onto silicon wafers, drying, and mounting onto SEM stubs under low vacuum conditions to ensure proper alignment with the electron beam.

#### Nitrogen adsorption-desorption isotherms (BET)

Nitrogen adsorption-desorption isotherms, following the Brunauer-Emmett-Teller (BET) method, were utilized to examine the pore structure and specific surface area of MPDA. This method measures nitrogen gas adsorption on the material’s surface at various relative pressures to determine pore size distribution and surface area. Before analysis, MPDA powder was degassed to remove surface and pore adsorbed gases. Nitrogen adsorption experiments were performed at controlled relative pressures, and the data were analyzed using BET and Barrett-Joyner-Halenda (BJH) theories to calculate specific surface area, pore volume, and pore size distribution.

#### Zeta potential and hydrodynamic size measurements

Zeta potential and hydrodynamic size were assessed using dynamic light scattering (DLS). CeO₂, MPDA, macrophage membrane-derived nanovesicles (MM NVs), and M-MPDA@CeO₂ were each dispersed in suitable buffers to maintain homogeneity and reduce aggregation. The instrument was calibrated, and all system components, including the light source and detectors, were confirmed to be functioning optimally. Samples were placed in measurement cells, with parameters such as temperature and equilibration time configured before data collection. Three independent replicates were conducted for each sample to ensure statistical reliability.

#### Stability evaluation of M-MPDA@CeO₂

To assess the stability of M-MPDA@CeO₂, 2 mg of the nanocomposite was dispersed in 5 mL of DMEM, FBS, or PBS and incubated at 37 °C for one week. Aliquots were collected, and dynamic light scattering (DLS) was used to measure the hydrodynamic size, thereby evaluating the impact of different media on particle aggregation.

#### X-ray photoelectron spectroscopy (XPS)

XPS, a surface-sensitive technique, analyzes the elemental composition and chemical states of materials by using X-rays to eject photoelectrons from the sample surface. The kinetic energy and intensity of these photoelectrons provide elemental and bonding information. In this study, M-MPDA@CeO₂ was examined using an XPS spectrometer with a monochromatic Al Kα X-ray source (1486.6 eV). The X-ray beam was directed at the sample surface, and the emitted photoelectrons were collected to produce high-resolution spectra for detailed surface characterization.

#### Ultraviolet-Visible (UV-Vis) spectroscopy

UV-Vis spectroscopy was utilized to examine the absorption characteristics of CeO₂, MPDA, and M-MPDA@CeO₂ in the ultraviolet and visible spectra. These absorption bands, resulting from electronic transitions between energy levels, offer insights into molecular structure, concentration, and chemical states. Samples were diluted with ultrapure water, sonicated for homogeneity, and placed in quartz cuvettes. Absorption spectra were recorded using a UV-Vis spectrophotometer over a wavelength range of 200–800 nm.

#### Fourier transform infrared (FT-IR) spectroscopy

FT-IR spectroscopy, utilizing Fourier transform principles, facilitates the rapid acquisition of high-resolution infrared spectra with improved sensitivity. Dried CeO₂, MPDA, and M-MPDA@CeO₂ powders were mixed with KBr, finely ground, and pressed into transparent pellets. FT-IR spectra were obtained in the range of 400–400 cm⁻¹ to identify functional groups and chemical interactions in the samples.

#### CeO₂ release profile from M-MPDA@CeO₂

CeO₂ release depends on the nanocomposite’s structural breakdown. Morphological changes in M-MPDA@CeO₂ after 2 h in 1 mM H₂O₂ were examined using scanning electron microscopy (SEM). To quantify CeO₂ release under simulated inflammatory conditions, 1 mg of M-MPDA@CeO₂ was digested overnight in aqua regia (HNO₃/HCl, 1:3 v/v) and diluted to 5 mL. The Ce content was measured using inductively coupled plasma optical emission spectrometry (ICP-OES) (*n* = 3). For sustained release simulation, 5 mg of M-MPDA@CeO₂ was dispersed in 10 mL of PBS with or without 1 mM H₂O₂ and incubated at 37 °C with continuous shaking. At specific intervals, 1 mL of suspension was centrifuged, and the supernatant was collected. Fresh PBS was added to maintain volume. The supernatants were digested with aqua regia, diluted to 5 mL, and analyzed via ICP-OES (*n* = 3) to develop the CeO₂ release profile.

#### ROS scavenging capacity of M-MPDA@CeO₂

Reactive oxygen species (ROS), such as superoxide radicals (O₂·⁻), hydroxyl radicals (·OH), and hydrogen peroxide (H₂O₂), are highly reactive molecules that cause oxidative damage by lipid peroxidation, leading to reactive lipid dicarbonyls. These byproducts disrupt cellular macromolecules, including membranes, proteins, and nucleic acids.

##### Electron paramagnetic resonance (EPR)

EPR spectroscopy, which relies on the resonance absorption of unpaired electrons in a magnetic field, assessed the radical scavenging capacity of M-MPDA@CeO₂. The nanocomposite was incubated with 1 mM H₂O₂ and spin-trapping agents (DMPO/BMPO) to form stable adducts with ·OH and O₂·⁻. EPR signals were recorded at specific intervals under optimized conditions to quantify radical scavenging.

##### Catalase (CAT)-like activity assay

The CAT-mimetic activity of M-MPDA@CeO₂ was evaluated by measuring O₂ production from H₂O₂ decomposition. M-MPDA@CeO₂ solutions (50–200 µg/mL) were combined with 1 mM H₂O₂ in a total volume of 5 mL and gently stirred. Dissolved oxygen levels were recorded every minute for 15 min using a dissolved oxygen meter.

##### TMB chromogenic reaction

The ·OH scavenging capacity was assessed using the TMB/H₂O₂ system. A combination of 100 µL M-MPDA@CeO₂ (50–250 µg/mL), 3 mL H₂O₂ (1 mM), and 100 µL TMB (0.8 mmol/L) was incubated at 37 °C for 5 min. The absorbance of the oxidized TMB was recorded at 550–750 nm with a UV-Vis spectrophotometer.

### Cell subculture

Once cell confluence reached 70–80%, the culture flask was removed from the incubator. Cells were washed twice with PBS and detached with 1 mL of 0.25% trypsin at 37 °C for 2 min. Digestion was halted by adding 3 mL of fresh complete medium. The cell suspension was centrifuged at 100 rpm for 5 min in a 15 mL tube. After discarding the supernatant, the pellet was resuspended in 3 mL of fresh medium. A 1 mL aliquot was transferred to a new culture flask, supplemented with 3 mL of medium, and incubated at 37 °C with 5% CO₂.

### Cytotoxicity assay of M-MPDA@CeO₂ on MLE-12 and RAW264.7 cells

Cells (1 × 10⁴/well) were seeded in 96-well plates and incubated overnight. The medium was then replaced with 100 µL of fresh medium containing M-MPDA@CeO₂ at concentrations of, 12.5, 25, 50, 100, or 200 µg mL⁻¹. After 24 h, the medium was removed, and the cells were washed once with PBS. Subsequently, 100 µL of serum-free medium with 10% CCK-8 reagent was added to each well and incubated at 37 °C for 1 h. Absorbance at 450 nm was measured to assess cell viability.

### CCK-8 assay for oxidative damage model

MLE-12 cells (1 × 10⁴/well) were exposed to 1 µg/mL LPS for 12 h to induce oxidative damage. Following PBS washing, cells were treated with M-MPDA@CeO₂ (–300 µg/mL) for 24 h. Cell viability was measured using CCK-8, as detailed in Sect. “Cytotoxicity assay of M-MPDA@CeO₂ on MLE-12 and RAW264.7 cells”.

### Transmission electron microscopy (TEM) of cells

#### Sample preparation

MLE-12 cells (5 × 10⁵/dish) were subjected to the following treatments: Control, LPS (1 µg/mL), LPS + MPDA, LPS + MPDA@CeO₂, and LPS + M-MPDA@CeO₂. After 12 h, cells were trypsinized, centrifuged at 100 rpm for 5 min, and fixed with 3% glutaraldehyde for 5 min at 4 °C. The fixed cells were then transferred to 1.5 mL microcentrifuge tubes and pelleted by centrifugation at 12,000 rpm for 10 min.

#### Fixation and dehydration

Cells were post-fixed with 1% osmium tetroxide, dehydrated in a graded acetone series (30% → 100%), and infiltrated with Epon812 resin (3:1, 1:1, and 1:3 ratios).

#### Embedding and sectioning

Samples were embedded in Epon812, and ultrathin Sects.  (60–90 nm) were cut using an ultramicrotome. Sections were mounted on copper grids.

#### Staining and imaging

Sections were stained with uranyl acetate (10–15 min) and lead citrate (1–2 min), then imaged using a JEM-1400FLASH TEM at 8000× magnification.

### Live/dead cell staining

MLE-12 cells (2 × 10⁴ per well) were treated according to experimental groups: Control, LPS, LPS+MPDA, LPS + MPDA@CeO₂, and LPS + M-MPDA@CeO₂. After 12 h, cells were stained with 500 µL of a live/dead staining solution containing 5% calcein-AM and 2% propidium iodide for 30 min at 37 °C. Following PBS washing, fluorescence images were captured.

### Mitochondrial membrane potential (JC-1 assay)

MLE-12 cells (2 × 10⁴ per dish) were treated and stained with JC-1 dye following the manufacturer’s instructions. After a 20-minute incubation at 37 °C, the cells were washed with JC-1 buffer and imaged using fluorescence microscopy to evaluate mitochondrial depolarization by measuring the red/green fluorescence ratio.

### Cellular uptake of nanocomposites

#### Fluorescence tracking

MLE-12 cells were incubated with Cy5.5-labeled M-MPDA@Cy5.5 (0–3 h), fixed, and counterstained with DAPI. Intracellular Cy5.5 fluorescence (red) was visualized using fluorescence microscopy.

#### ICP-OES quantification

Cells (7 × 10⁵/well) treated with M-MPDA@CeO₂ (200 µg/mL) were collected at 0.5–24 h, digested with aqua regia, and analyzed via ICP-OES to quantify Ce uptake (*n* = 3).

### Intracellular ROS detection

MLE-12 cells (5 × 10⁴ per well) were treated according to experimental conditions for 12 h. Cells were incubated with DCFH-DA (1:100 dilution) in basal medium for 20 min, shielded from light. Following washing, ROS levels were measured using fluorescence microscopy or flow cytometry (excitation/emission: 480/525 nm).

### Flow cytometric analysis of cell surface markers

Macrophages from experimental groups were harvested with EDTA-free trypsin, washed with PBS, and resuspended in staining buffer. Cells were then incubated with FITC-conjugated CD86 antibody (M1 marker) and PE-conjugated CD206 antibody (M2 marker) at a 1:400 dilution for 20 min on ice, protected from light.

(2) Cells were washed twice with staining buffer and centrifuged at 1000 rpm for 5 min.

(3) The pellet was resuspended in staining buffer and analyzed using a flow cytometer.

### Experimental animals

Six-week-old male BALB/c mice were obtained from Beijing Vital River Laboratory Animal Technology Co., Ltd. and maintained in a specific pathogen-free facility under a 12-hour light/dark cycle with unrestricted access to food and water. The study protocols received approval from the Ethics Committee of the Second Affiliated Hospital of Guangxi Medical University (Approval No.: 2021-KY0735).

### Hemocompatibility assay of M-MPDA@CeO₂

To assess the hemolytic potential of M-MPDA@CeO₂, whole blood from healthy mice was collected into anticoagulant tubes. The blood was centrifuged at 100 rpm for 5 min to isolate red blood cells (RBCs), which were then washed three times with PBS and resuspended in PBS to create a 2% RBC suspension. Equal volumes (500 µL) of M-MPDA@CeO₂ solutions (50–300 µg/mL) were mixed with the RBC suspension. PBS and ddH₂O were used as negative and positive controls, respectively. The mixtures were incubated at 37 °C for 1 h, centrifuged at 300 rpm for 15 min, and photographed. Supernatants (100 µL) were transferred to a 96-well plate, and absorbance at 542 nm was measured using a microplate reader (*n* = 3). Hemolysis percentage was calculated as follows:

### Blood biochemistry and body weight monitoring

The randomization method we used involved numbering the mice and then assigning them to groups using a random number table. To evaluate the in vivo safety of M-MPDA@CeO₂, 10 healthy BALB/c mice were randomly assigned to two groups (*n* = 5 per group). One group received daily tail vein injections of M-MPDA@CeO₂ (10 mg/kg, equivalent to 0.634 mg/kg Ce) for 14 days, while the control group was administered PBS. Body weight was monitored every two days. On day 14, blood samples were collected via retro-orbital puncture. Complete blood counts (WBC, RBC, HGB, PLT) and serum biochemical markers (ALT, AST, BUN, CREA) were measured using automated analyzers. Statistical comparisons were made with the control group.

### ALI mouse model

Mice were anesthetized with intraperitoneal pentobarbital sodium and positioned at a 45° tilt for intratracheal instillation of LPS (10 mg/kg in saline) to induce ALI. Control mice received saline. Post-instillation, mice were allowed to recover spontaneously.

### In vivo targeting study

ALI mice received an intravenous injection of M-MPDA@Cy5.5 or MPDA@Cy5.5 (200 µL, 200 µg/mL). Fluorescence signals were tracked at 0.5, 2, 4, 6, 8, 12, and 24 h post-injection using an in vivo imaging system. At 24 h, major organs (heart, liver, spleen, lung, kidney) were excised for ex vivo fluorescence analysis.

### Pharmacokinetic study

Three BALB/c mice received an intravenous dose of M-MPDA@CeO₂ (5 mg/kg). Blood samples (20 µL) were drawn from the retro-orbital sinus at intervals of 0.5, 1, 2, 4, 6, 12, 24, 36, 48, and 72 h. These samples were digested with aqua regia (HNO₃/HCl, v/v = 1:3) and analyzed using ICP-OES to measure Ce concentrations (*n* = 3).

### Therapeutic efficacy in ALI

Twenty-five male BALB/c mice were allocated into five groups (*n* = 5): Control, LPS, LPS+MPDA, LPS+MPDA@CeO₂, and LPS + M-MPDA@CeO₂. ALI was induced as described in Sect.  2.21. Two hours after LPS administration, treatment groups received 200 µL of nanoparticles (5 mg/kg) via tail vein injection, while the Control and LPS groups were given PBS.

### Bronchoalveolar lavage fluid (BALF) analysis

Mice were anesthetized, and bronchoalveolar lavage fluid (BALF) was collected by instilling 0.8 mL of ice-cold PBS three times, achieving a recovery rate of over 60%. The BALF was centrifuged, and the supernatants were stored at -80 °C. Total cell counts, protein concentration (via BCA assay), and cytokine levels (TNF-α, IL-1β, IL-6; determined by ELISA) were analyzed.

### Lung wet/dry weight ratio

Lungs were weighed (wet weight, W), dried at 70 °C for 72 h, and reweighed (dry weight, D). The ratio was calculated as W/D.

### Peripheral blood treg cell analysis

Regulatory T cells (Tregs), essential for immune balance and lung injury repair, were quantified. Blood samples underwent staining with anti-CD25-FITC and anti-Foxp3-PE antibodies at 4 °C, following manufacturer-recommended concentrations. Cells were then fixed, permeabilized using eBioscience™ buffers, washed, and analyzed by flow cytometry to identify CD25⁺Foxp3⁺ Tregs.

### Survival study

Forty ALI mice (LPS: 50 mg/kg, lethal dose) were randomized into LPS and LPS + M-MPDA@CeO₂ groups (*n* = 20/group). At 2 h post-LPS, the treatment group received 200 µL of M-MPDA@CeO₂ intravenously. Survival was monitored for 14 days (1: death, 0: survival).

### Lung histopathology

Lungs were fixed in 4% paraformaldehyde, embedded in paraffin, sectioned, and stained with hematoxylin and eosin for inflammatory evaluation. Apoptotic cells were identified using a TUNEL kit and observed through fluorescence microscopy. For malondialdehyde (MDA) immunohistochemistry, paraffin sections underwent antigen retrieval, endogenous peroxidase blocking, and incubation with an anti-MDA antibody. Visualization was achieved with DAB staining and hematoxylin counterstaining. Image analysis and histological scoring were performed by blinded observers according to the standardized scoring system. For blinding, the grouping, experimentation, and outcome assessment were carried out by three different researchers from our team. Both the mouse groups and the drug solutions were coded with numbers. We made every effort to adhere to the 3R principles (Replacement, Reduction, Refinement) in animal use, and the prerequisites for parametric statistical tests were satisfied. Frozen sections for ROS staining were treated with an autofluorescence quencher, stained with a ROS probe and DAPI, and examined via fluorescence microscopy.

### Statistical analysis

Data are presented as mean ± SD. Student’s t-test was employed for two-group comparisons, and one-way ANOVA was used for analyses involving multiple groups (Origin 2021). A P-value of less than 0.05 was considered statistically significant.

## Results

### Characterization of M-MPDA@CeO_2_

**Fig. 1:**
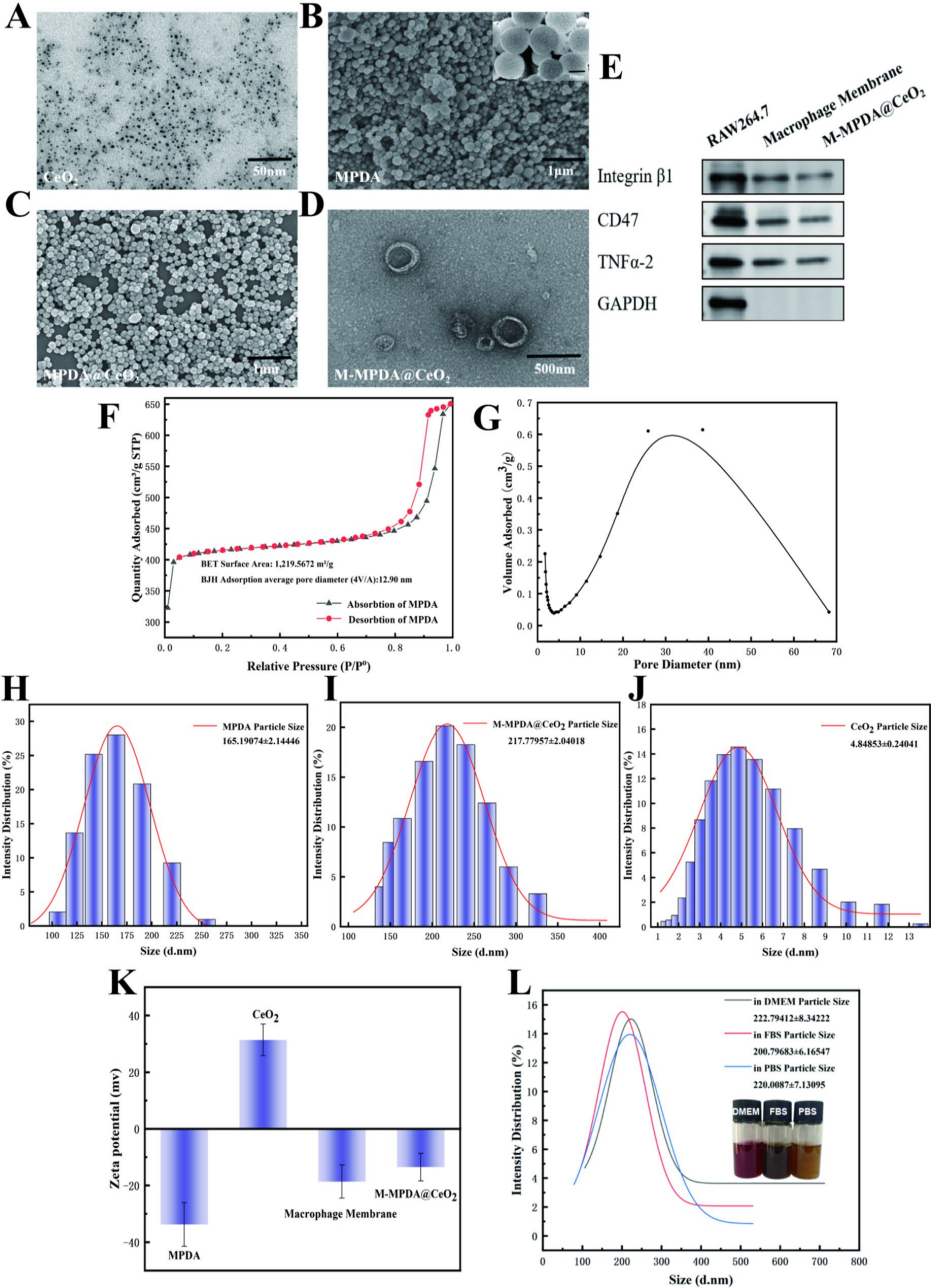
Synthesis and characterization of functionalized M-MPDA@CeO_2_. (**A**) The representative TEM images of CeO_2_(scale bar: 50 nm); (**B**) The representative SEM images of MPDA(scale bar: 1 μm); (**C**) The representative SEM images of MPDA@CeO_2_(scale bar: 1 μm); (**D**) The representative TEM images of M-MPDA@CeO2(scale bar: 500 nm); (**E**) Representative Western blot analysis of Integrin β1, CD47 and TNF-2 in RAW264.7, macrophage cell membrane microcapsule, and M-MPDA@CeO_2_; (**F**) Nitrogen adsorption (desorption) isotherms (BET) for MPDA; (**G**) MPDA aperture analysis; (Fig. 2. **H**-**J**) Particle size diagram of CeO_2_, MPDA@CeO_2_, M-MPDA@CeO_2_; (Fig. 2. **K**) Zeta potential of MPDA, CeO_2_, macrophage cell membrane microcapsule, M-MPDA@CeO_2_; (**L**) Structural Stability of M-MPDA@CeO_2_ in different solutions (DMEM, FBS, PBS)

The transmission electron microscopy (TEM) images show that the synthesized CeO_2_ consist of uniformly dispersed ultra-small dot-like nanoparticles with a particle size of approximately 5 nm (Fig. 1A). The MPDA presents as uniformly distributed, monodisperse spherical structure with a particle size of approximately 200 nm (Fig. 1B). After loading CeO_2_, MPDA@CeO_2_ still exhibits uniformly sized spherical structures with good dispersibility, and the absorption of CeO_2_ can be clearly observed on the outer surface of MPDA, confirming that CeO_2_ can be successfully loaded into the MPDA structure (Fig. 1C). To verify the successful coating of macrophage membranes onto MPDA@CeO_2_, the microstructure of M-MPDA@CeO_2_ was first observed using TEM. The results reveal a distinct single-layer light-colored coating on the outer surface of M-MPDA@CeO_2_, with clear boundaries displaying an obvious core-shell structure, which is presumed to be the outer layer of coated macrophage membranes (Fig. 1D).

**Fig. 2:**
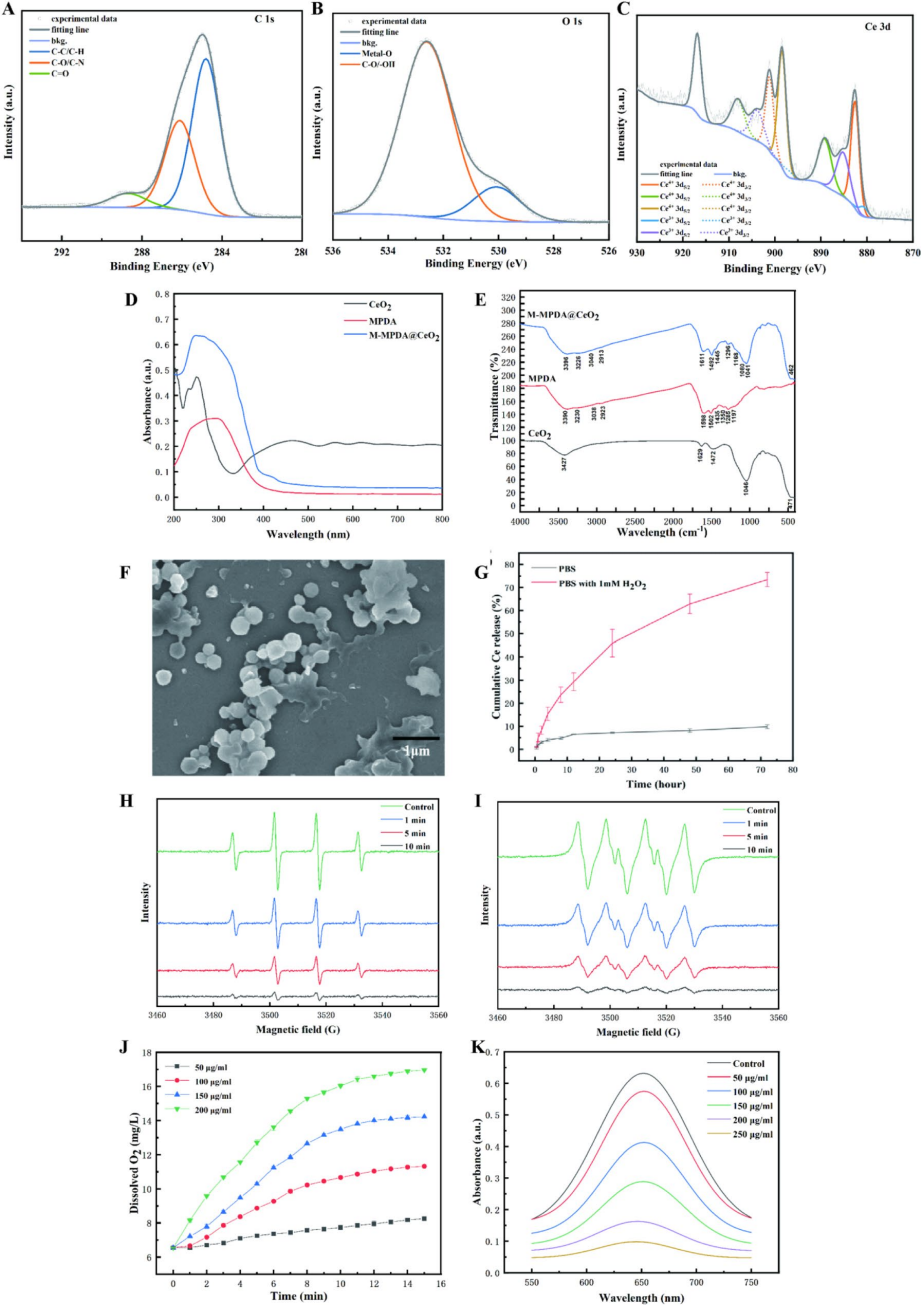
Synthesis of functionalized M-MPDA@CeO_2_. X-ray photoelectron spectrum at M-MPDA@CeO_2_.(**A**)C;(**B**)O;(**C**)Ce.(**D**) Ultraviolet absorption spectra of CeO_2_, MPD, M-MPDA@CeO_2;_(**E**)FT-IR spectra of CeO_2_, MPDA, and M-MPDA@CeO_2_. (**F**) SEM morphology of M-MPDA@CeO_2_ pyrolysis in hydrogen peroxide solution; (**G**) Release curve of Ce for M-MPDA@CeO_2_ in hydrogen peroxide solution. (**H**-**I**) The antioxidant capacity of M-MPDA@CeO_2_ to remove ·OH and O_2_·^−^ determined by ESR; (**J**) The simulated activity of CAT enzyme M-MPDA@CeO_2_ catalyzes hydrogen peroxide to produce oxygen(0;50;100;150;200 µg/mL); (**K**) UV-VIS absorption spectra of M-MPDA@CeO_2_ after incubation of TMB+·OH at different concentration gradients(0;50;100;150;200;250 µg/mL)

Since macrophages rely on functional proteins on their membranes to achieve homing effects to inflammatory sites, western blot was used to analyze the expression of key functional proteins, including integrin β1, CD47, and TNFα-2, in RAW264.7 cells, macrophage membrane vesicles, and M-MPDA@CeO_2_ (Fig. 1E). The results demonstrated that compared with RAW264.7 cells, both macrophage membrane vesicles and M-MPDA@CeO_2_ contained the aforementioned functional proteins expressed by RAW264.7 cells, indicating that these membrane proteins remained present after physical extrusion. GAPDH, a typical cytoplasmic secretory protein, was not expressed in macrophage membrane vesicles or M-MPDA@CeO_2_, confirming that the extracted macrophage membrane vesicles are pure.

Analysis on the nitrogen adsorption-desorption isotherm of MPDA reveals a specific surface area of 1219.57 m²/g as calculated by the BET method, and an average pore diameter of approximately 12.90 nm determined by the BJH method (Fig. 1F and G). The high specific surface area and porous structure of MPDA suggests its strong potential for loading small-molecular substances, making it suitable for applications such as drug carriers. The particle size distributions of CeO_2_, MPDA, and M-MPDA@CeO_2_ are shown in Fig. 1H and J. The results manifest that the average particle size of CeO_2_ is 4.85 ± 0.24 nm, that of MPDA is approximately 165.19 ± 2.14 nm, and that of M-MPDA@CeO_2_ is approximately 217.78 ± 2.04 nm. Comparison reveals that the particle size of M-MPDA@CeO_2_ increases significantly after macrophage membrane coating. The zeta potential data are shown in Fig. 1K. The zeta potentials of CeO_2_ are 31.40 ± 5.61 mV, that of MPDA is -33.77 ± 7.73 mV, that of macrophage membrane vesicles is -18.63 ± 5.89 mV, and that of M-MPDA@CeO_2_ is -13.51 ± 4.87 mV. Analysis of the zeta potential demonstrates that after coating with the macrophage membrane, the positive charge of CeO_2_ is shielded, and M-MPDA@CeO_2_ exhibits a potential close to that of the macrophage membrane. These findings confirm that macrophage membrane is successfully coated on the outer surface of M-MPDA@CeO_2_. The macrophage membrane camouflage helps M-MPDA@CeO_2_ mimic the functions of macrophages, laying the foundation of subsequent experiments.

The stability of nanomaterials is crucial for ensuring the sustainability, safety, and superior performance of their applications as biologics. The stability of M-MPDA@CeO_2_ was preliminarily evaluated by measuring its intensity distribution in different solutions. We observed that after standing for one week in DMEM and PBS, the particle sizes of M-MPDA@CeO_2_ were 222.79 ± 8.34 nm and 220.01 ± 7.13 nm, respectively, with minimal changes, indicating good stability and uniform distribution (Fig. 1L). In FBS, the particle size was 200.80 ± 6.17 nm, slightly smaller than in DMEM and PBS, though the overall difference was not significant. This slight reduction may be attributed to the complex components of serum proteins interacting with the surface of M-MPDA@CeO_2_, leading to changes in surface charge. Such charge alterations could affect the interaction forces between nanoparticles, resulting in a slightly smaller particle size measured in FBS.

### Performance evaluation of M-MPDA@CeO_2_

Analyses by XPS indicate that characteristic peaks for C and O appear at the corresponding binding energy positions of C1s and O1s in M-MPDA@CeO_2_, confirming the presence of C and O elements in the material (Fig. 2A and C). In the Ce3d spectrum of M-MPDA@CeO_2_, Ce exhibits both Ce^3+^ (880.84, 885.16, 897.16, and 903.88 eV) and Ce⁴⁺ (882.56, 889.13, 898.43, 901.13, 908.03, and 916.82 eV) states. The characteristic elemental peaks detected by XPS verify the existence of C, O, and Ce in the final product M-MPDA@CeO₂, while also confirming the coexistence of both valence states of Ce, where Ce³⁺ is the reduced state and Ce⁴⁺ is the oxidized state. Since the enzyme-like activity of CeO₂ depends on the ratio of Ce³⁺ to Ce⁴⁺, we further quantified this ratio. In M-MPDA@CeO₂, the ratio of Ce^3+^ to Ce^4+^ is 21.5: 78.49. Ce^3+^ serves as the reduced active site in CeO₂, capable of absorbing, storing, and releasing oxygen. Its redox cycle enables CeO₂ to absorb oxygen and form Ce⁴⁺, while under reducing conditions, Ce⁴⁺ can be reduced back to Ce³⁺. Additionally, the presence of oxygen vacancies in CeO₂ enhances its surface activity.

To confirm the successful synthesis of M-MPDA@CeO_2_, UV-Vis and FT-IR characterizations were performed. The UV-Vis absorption spectrum results were shown in Fig. 2D. CeO_2_ exhibits an absorption peak at 251 nm, while MPDA displays a typical characteristic absorption peak at 280 nm, which corresponds to the π-π electronic transition of the aromatic ring in dopamine. The final product, M-MPDA@CeO_2_, displays the same characteristic absorption peak near 250 nm as that of CeO_2_. Typically, the UV absorption peak of CeO_2_ occurs at shorter ultraviolet wavelengths, specifically in the ultraviolet region. In the spectrum of CeO₂, the exact position and shape of the absorption peak can be influenced by factors such as the crystal structure, morphology, and the presence of oxygen vacancies.

Furthermore, the FT-IR structural qualitative analysis of the samples was conducted. The infrared spectrum of CeO_2_ exhibits a peak at 1629 cm^− 1^ attributed to the O-H bending vibration of water, peaks at 1472 cm^− 1^ and 1046 cm^− 1^ introduced by other impurities in the CeO_2_ sample (C-O stretching vibrations from the solvent), and a peak at 471 cm^− 1^ corresponding to the Ce-O stretching vibration of CeO_2_ (characteristic peak, Fig. 2E). In the spectrum of MPDA, peaks are observed at 3390 cm^− 1^ (O-H bending vibration of water), 3230 cm^− 1^ (N-H stretching vibration), 3038 cm^− 1^ (C-H stretching vibration of the aromatic ring), 2923 cm^− 1^ (C-H stretching vibration of methylene groups), 1611 cm^− 1^ and 1492 cm^− 1^ (C = C stretching vibrations of the aromatic ring), 1445 cm^− 1^ (C-H out-of-plane bending vibration of methylene and methyl groups), 1350 cm^− 1^ (C-N stretching vibration of aromatic amines), 1285 cm^− 1^ (C-O stretching vibration of phenols), and 1197 cm^− 1^ (C-N stretching vibration of aliphatic amines). In the FT-IR spectrum of M-MPDA@CeO_2_, peaks are observed at 3396 cm^− 1^ (O-H bending vibration of water), 3226 cm^− 1^ (N-H stretching vibration), 3040 cm^− 1^ (C-H stretching vibration of the aromatic ring), 2913 cm^− 1^ (C-H stretching vibration of methylene groups), 1598 cm^− 1^ and 1502 cm^− 1^ (C = C stretching vibrations of the aromatic ring), 1435 cm^− 1^ (C-H out-of-plane bending vibration of methylene groups), 1350 cm^− 1^ (C-N stretching vibration of aromatic amines), 1285 cm^− 1^ (C-O stretching vibration of phenols), 1168 cm⁻¹ (P = O stretching vibration of phospholipids on the cell membrane), 1085 cm^− 1^ (C-O stretching vibration of esters on the cell membrane), and 462 cm^− 1^ (Ce-O stretching vibration of CeO₂). These analytical results confirm the successful synthesis of M-MPDA@CeO_2_.

SEM images showed that after treatment with 1mM H_2_O_2_ for 2 h, M-MPDA@CeO_2_ exhibited significant morphological degradation (Fig. 2F). The disintegration characteristics of M-MPDA@CeO_2_ in H_2_O_2_ led to the responsive release of CeO_2_. ICP-OES detection results indicated that the Ce content in M-MPDA@CeO_2_ was 6.34%. The cumulative release rate of Ce in a solution containing 1 mM H_2_O_2_ reached 73.41% over 72 h, whereas in PBS, the cumulative release rate over the same period was only 9.79% (Fig. 2G). The morphological degradation of M-MPDA@CeO₂ and the release of CeO₂ under H₂O₂ conditions demonstrate the high sensitivity of the nanocomposite to ROS and its controllable release characteristics.

To systematically investigate the potent ROS scavenging capability of M-MPDA@CeO_2_, we focused on representative ROS species:·OH, O2·⁻, and H_2_O_2_. Employing DMPO to capture ·OH and form the DMPO/·OH spin adduct, compared to the control group without M-MPDA@CeO_2_, the ESR signal intensity decreased over time after adding M-MPDA@CeO_2_. After 10 min of reaction, M-MPDA@CeO₂ almost completely scavenged •OH (Fig. 2H). This result verifies that M-MPDA@CeO₂ has a strong •OH scavenging ability. Employing BMPO as a spin trap for superoxide, the the results showed that the control group produced a strong ESR signal. After adding M-MPDA@CeO₂, the ESR signal intensity also significantly decreased with prolonged reaction time, demonstrating its antioxidant ability to scavenge superoxide (Fig. 2I). After reacting with H₂O₂, the amount of O₂ generated gradually increased with higher concentrations of M-MPDA@CeO₂ and extended reaction time (Fig. 2J), confirming that M-MPDA@CeO₂ exhibits excellent CAT-like catalytic performance.

The TMB chromogenic reaction was employed to evaluate the oxygen radical scavenging ability of M-MPDA@CeO₂. In the TMB-H₂O₂ reaction system, a typical chromogenic reaction and a characteristic absorption peak at 652 nm were rapidly generated. However, when M-MPDA@CeO₂ was added to the reaction system, the blue color of TMB gradually faded, and the absorbance at 652 nm decreased with increasing concentrations of M-MPDA@CeO₂ (Fig. 2K). This indicates that M-MPDA@CeO₂ can significantly scavenge oxygen radicals, thereby inhibiting the oxidation of TMB, and its scavenging ability is closely related to its concentration.

### In vitro evaluation on the antioxidant function and cellular uptake

**Fig. 3:**
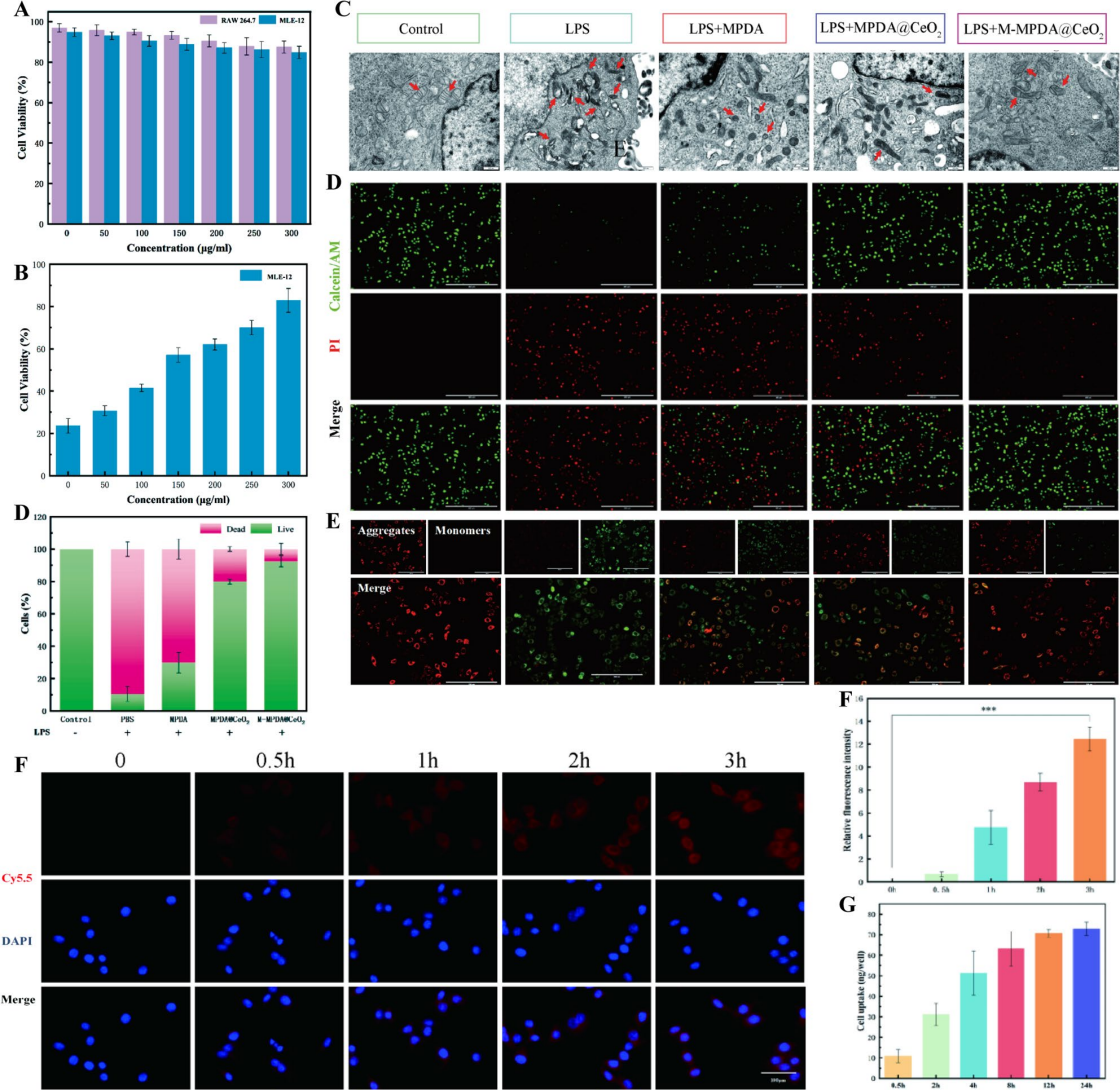
Investigation of Evaluation of the cellular uptake and biocompatibility of M-MPDA@CeO_2_ in RAW264.7 cells and MLE-12 cells. (**A**) Toxicity of M-MPDA@CeO_2_ to RAW264.7 cells and (**B**) MLE-12 cells; Effect of M-MPDA@CeO_2_ on the viability of LPS-stimulated MLE-12 cells; (**C**) Transmission electron microscopy images of MLE-12 cells after various intervention treatments. (Indicates mitochondria, Scale: 500 nm); (**D**) Calcein-AM /PI double staining images of MLE-12 cells after different intervention treatments and the fluorescence quantization (Scale: 400 μm); (**E**) Mitochondrial membrane potential changes of MLE-12 cells after different treatments (Scale: 200 μm); (**F**) Fluorescence microscope images of Cy5.5 uptake by MIE-12 cells at different times after co-incubation with M-MPDA@Cy5.5 solution (100 µg/mL). (Scale: 100 μm); (**G**) Fluorescence quantization map of uptake of M-MPDA@Ce6 by MIE-12 cells (*P* < 0.001)

As a novel nanocomposite designed for drug delivery, the effects of M-MPDA@CeO₂ on cytotoxicity, protection against oxidative stress, and the polarization of macrophages were evaluated. CCK-8 assay was conducted to assess the impact of M-MPDA@CeO_2_ on cell viability. After treating cells with M-MPDA@CeO₂ at concentrations up to 200 µg/mL, no significant cytotoxicity was observed in either cell line, with cell viability remaining around 90% (Fig. 3A). Therefore, 200 µg/mL was selected as the working concentration for M-MPDA@CeO₂ in this experiment. LPS, as a potent extracellular stimulant, can induce oxidative stress in MLE-12 cells, leading to damage in both cellular structure and function. LPS treatment significantly reduced cell viability, increasing concentrations of M-MPDA@CeO_2_ facilitated recovery, suggesting a protective role against LPS-induced oxidative damage (Fig. 3B).

TEM was used to observe the morphological and structural changes in mitochondria of LPS-induced MLE-12 cells under different treatments (Fig. 3C). In the control group, mitochondria exhibited normal morphology and structure, appearing nearly elliptical in shape with clear and straight cristae, and uniform gray matrix electron density. After LPS induction, mitochondrial morphology and structure showed significant abnormalities. A large number of mitochondria in the cytoplasm appeared markedly condensed and reduced in volume, taking on a thin rod-like or spherical shape, with reduced or fragmented cristae, significantly widened or even ruptured intra-cristae spaces, and darkened matrix electron density. Following treatment with MPDA, MPDA@CeO₂, and M-MPDA@CeO₂, the degree of mitochondrial damage gradually decreased compared to the model group. Notably, in the M-MPDA@CeO₂ group, only mild mitochondrial swelling was observed, with the structure closely resembling that of normal mitochondria. Living and dead cells were labeled using Calcein/PI, and the results were shown in Fig. 3D. Compared with the control group, the number of living cells in the model group significantly decreased. After treatment with MPDA, MPDA@CeO₂, and M-MPDA@CeO₂, respectively, the number of dead MLE-12 cells gradually decreased, while the number of living cells gradually increased.

Mitochondrial membrane potential (MMP) is the potential difference across the mitochondrial membrane within cells. It is one of the key factors maintaining mitochondrial function and energy production, playing an important role in cellular metabolism, survival, and apoptosis. The changes in mitochondrial membrane potential under different treatment methods are shown in Fig. 3E. Compared with the control group, the red fluorescence signal in the model group almost disappeared. However, after the model group cells were treated with MPDA, MPDA@CeO₂, and M-MPDA@CeO₂, respectively, the red fluorescence signal gradually increased while the green fluorescence signal gradually weakened, indicating that the addition of the nanomaterials partially restored the damaged mitochondria. Most notably, in the model group treated with M-MPDA@CeO₂, we observed a significant enhancement of the red fluorescence signal and a reduction of the green fluorescence signal, demonstrating that M-MPDA@CeO₂ effectively repairs LPS-induced mitochondrial damage in MLE-12 cells and protects the cells from oxidative stress-induced apoptosis.

In this study, the viability of MLE-12 cells significantly decreased after LPS treatment, and abnormal mitochondrial morphology was observed, including volume shrinkage, cristae fragmentation, and increased electron density, which are the direct evidence of cellular damage. As the energy powerhouse of the cell, the structural and functional integrity of mitochondria is crucial for maintaining cell survival. A decrease in mitochondrial membrane potential typically reflects impaired mitochondrial function, which further leads to disturbances in energy metabolism and cell death. The above experimental results indicate that MPDA exhibits certain antioxidant effects, while MPDA@CeO₂ demonstrates synergistic antioxidant functionality. M-MPDA@CeO₂ displayed the best antioxidant performance. We hypothesize that after modification with macrophage membranes, the phospholipid bilayer structure on the surface of MPDA@CeO₂ enhances cellular uptake of the nanomaterial, while its antioxidant function is positively correlated with the quality of the material.

To verify the uptake of the nanocomposite by MLE-12 cells under in vitro conditions, MLE-12 cells were co-incubated with M-MPDA@Cy5.5. Fluorescence microscopy observations at different time points revealed significant red fluorescence signals clustered around the nuclei of M-MPDA@Cy5.5-treated MLE-12 cells, with the fluorescence intensity increasing over time (Fig. 3F). This indicates that the uptake of the nanocomposite by MLE-12 cells is positively correlated with time. The Ce content per well in MLE-12 cells after uptake of M-MPDA@CeO₂ was quantitatively detected by ICP-OES. As shown in Fig. 3G, the Ce content per well gradually increased with prolonged incubation time. At 8 h of co-incubation, the total Ce content per well was approximately 63.09 ± 8.42 ng/Well. Beyond this time, the increase in Ce content was not significant, suggesting that the uptake of M-MPDA@CeO₂ by MLE-12 cells may have reached saturation.

### In vitro evaluation on ROS elimination and macrophage polarization

**Fig. 4:**
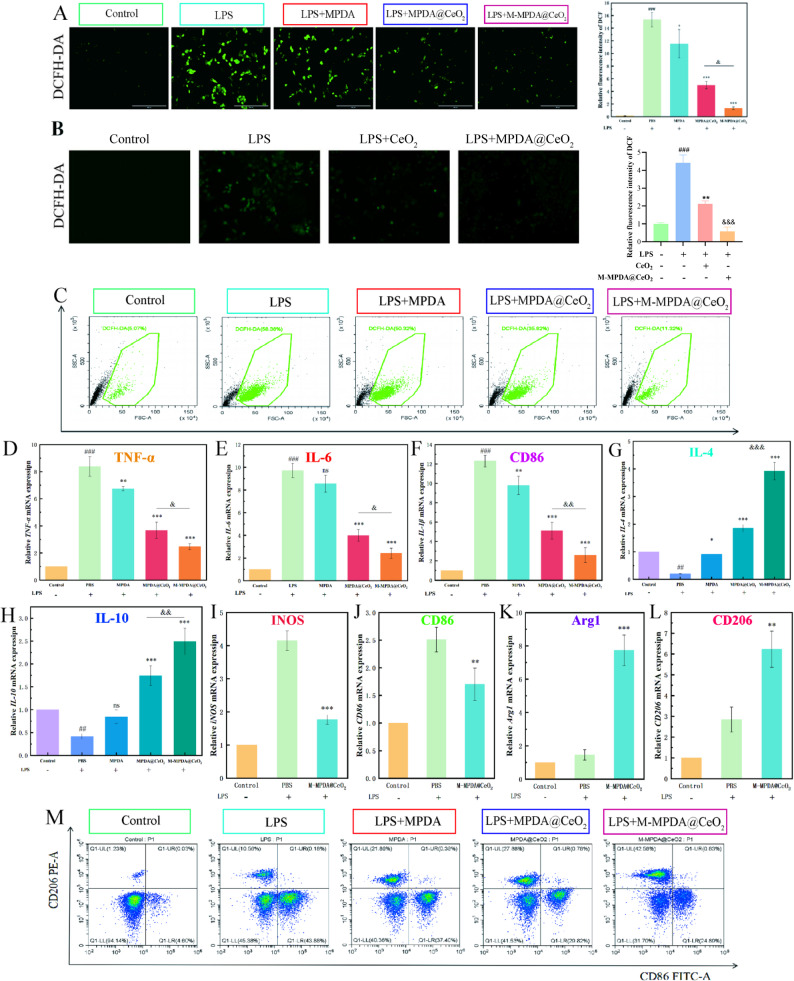
Investigation of Anti-oxidative damage and anti-inflammatory MLE-12cells and macrophage polarization properties in RAW264.7 cells. (**A**) Fluorescence microscope images of ROS clearance capacity of MLE-12 cells under different treatment conditions and fluorescence intensity quantization reslults (Scale: 400 μm); (**B**) Fluorescence microscope images of ROS clearance capacity of MLE-12 cells under different treatment conditions and fluorescence intensity quantization reslults; (**C**) Flow cytometry was used to detect ROS clearance of MLE-12 cells under different treatment conditions; (**D**-**F**) Relative mRNA levels of TNF-α(I), IL-1β(II) and IL-6(III) in RAW264.7 cells induced by LPS after different treatments; (**G**-**L**) The relative expression levels of IL-4, IL-10, iNOS, CD86, Arg1 and CD206 in RAW264.7 macrophages were detected by qRT-PCR (*n* = 3,**P < 0.01 vs. model group; ***P < 0.001vs model group).(**M**) Flow cytometry analysis of macrophage CD86 and CD206 expression.(*n* = 3, ###*P* < 0.001 vs. control group; *P < 0.05 vs. model group; ***P < 0.001vs model group; &*P* < 0.05)

The essence of ALI involves excessive production of ROS in the body, leading to uncontrolled inflammatory responses and excessive infiltration of inflammatory cells in the lungs. Thus, eliminating excessively produced ROS is an effective approach to alleviate ALI symptoms. To evaluate the ROS-scavenging ability of M-MPDA@CeO₂ at the cellular level, validation was performed using the classic ROS probe DCFH-DA through fluorescence microscopy imaging. As shown in Fig. 4A, compared to the control group, significant green fluorescence signals in the model group indicated excessive intracellular ROS production after LPS stimulation. As illustrated in Fig. 4B, at equivalent Ce doses, M-MPDA@CeO₂ exhibited significantly greater ROS scavenging capacity than free CeO₂. In the model group cells treated with MPDA, MPDA@CeO₂, and M-MPDA@CeO₂, respectively, the fluorescence signals gradually weakened. Compared to the MPDA@CeO₂ group, cells treated with M-MPDA@CeO₂ exhibited even weaker ROS fluorescence signals, with a statistically significant difference of *P* < 0.05. Flow cytometry analysis of ROS yielded results consistent with the fluorescence observations (Fig. 4C).

Macrophage polarization refers to the functional and phenotypic changes macrophages undergo under specific microenvironmental conditions, which can be classified into two states: M1 and M2. M1 macrophages are typically polarized by bacterial components or pro-inflammatory factors, exhibiting pro-inflammatory responses and cytotoxicity. Surface markers of M1 macrophages include CD86. In contrast, M2 macrophages are mainly influenced by cytokines such as IL-4 and IL-10, displaying anti-inflammatory responses, tissue repair, and immunomodulatory functions. Surface markers of M2 macrophages include CD206. After RAW264.7 cells were treated according to different experimental groups for 24 h, the expression of inflammatory genes TNF-α and IL-6 (Fig. 4D and E) in the model group was significantly upregulated (*P* < 0.001), and the gene expression of M1 macrophage marker CD86 was also upregulated (Fig. 4F). On the contrary, the anti-inflammatory cytokines IL-4 and IL-10 were significantly downregulated by LPS treatment (Fig. 4G and H). Following treatment with MPDA, MPDA@CeO₂, and M-MPDA@CeO₂, respectively, the expression levels of inflammatory cytokines in all groups decreased to varying degrees compared to the model group (*P* < 0.05), while the expression levels of anti-inflammatory cytokines was increased. The M-MPDA@CeO₂ group showed a more significant alteration in the expression of pro- or anti-inflammatory cytokine genes compared to the MPDA@CeO₂ group (*P* < 0.05). The upregulation of M1 macrophage markers (iNOS and CD86) induced by LPS was reversed by M-MPDA@CeO₂ treatment (Fig. 4I and J) (*P* < 0.05). The expression levels of M2 macrophage-related genes (Arg1 and CD206) were upregulated after intervention with M-MPDA@CeO₂ compared to the model group (Fig. 4K and L) (*P* < 0.05).

Flow cytometry was used to detect the expression of CD86 and CD206 to assess macrophage polarization. The results are shown in Fig. 4M. In the model group, the proportion of CD86-positive cells was significantly higher compared to other groups, indicating that LPS stimulation upregulated CD86 expression. After treatment with MPDA, MPDA@CeO₂, and M-MPDA@CeO₂, respectively, the proportion of CD86-positive cells decreased, while the proportion of CD206-positive cells gradually increased. The results indicate that both MPDA and MPDA@CeO₂ possess the ability to suppress CD86 expression and enhance CD206 expression to some extent, and that M-MPDA@CeO₂ can synergistically strengthened the ability to inhibit M1 macrophage polarization and promote M2 macrophage polarization. However, M-MPDA@CeO₂ treatment showed no similar polarization effect on bone marrow derived macrophages (Figure S1). The balance of macrophage polarization is closely associated with immune regulation, inflammation control, and tissue repair; hence its dysregulation may contribute to the development and progression of inflammatory and immune-related diseases. Therefore, investigating the regulatory mechanisms of macrophage polarization and related therapeutic strategies holds significant importance for disease prevention and treatment.

### Assessment on biocompatibility of M-MPDA@CeO_2_ in vitro and in vivo

**Fig. 5:**
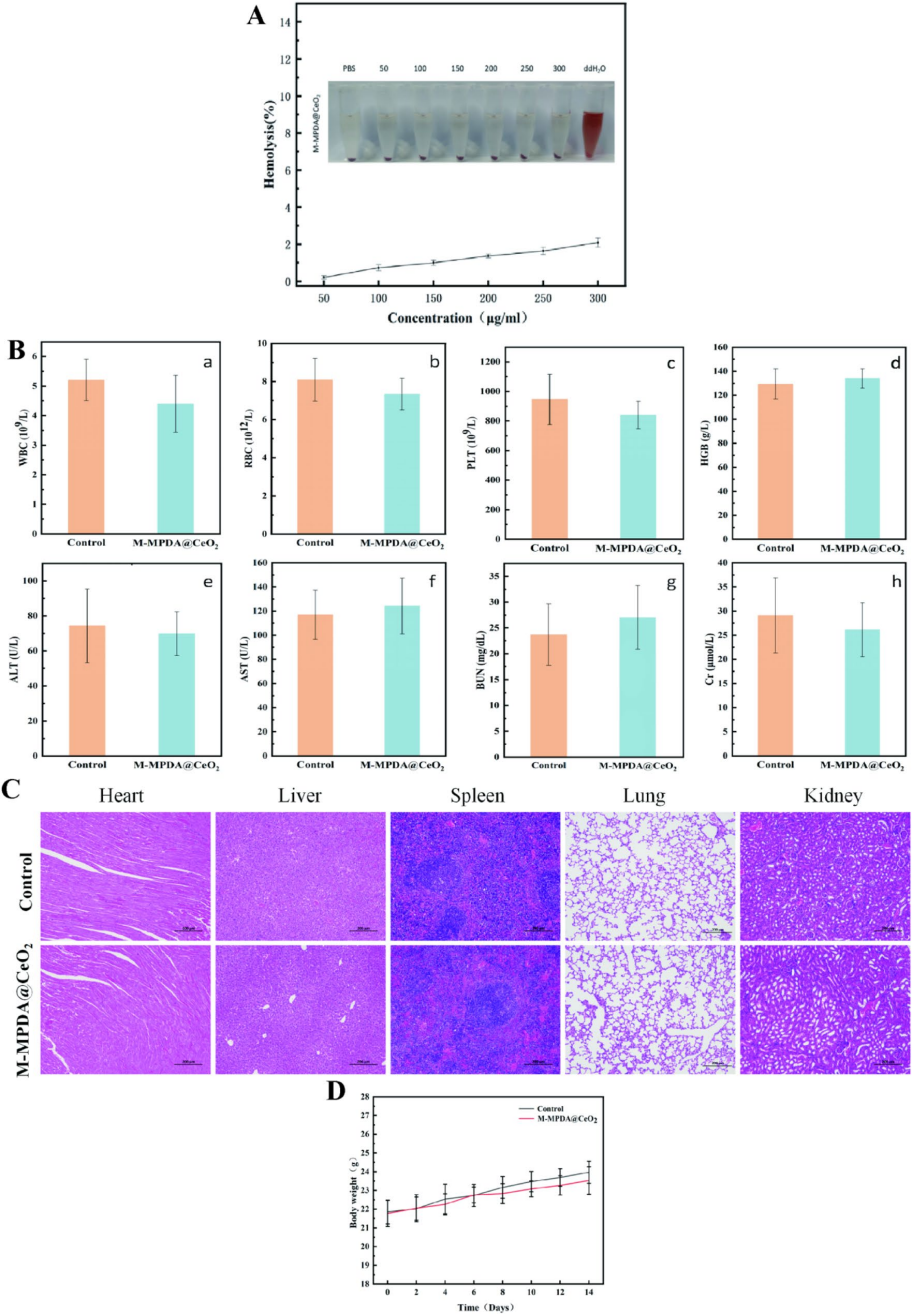
Biocompatibility of M-MPDA@CeO_2_ in vitro and in vivo. (**A**) Detection of hemolytic activity of red blood cells after coculture with various concentrations of M-MPDA@CeO_2_, the optical photograph showing different concentrations of M-MPDA@CeO_2_ groups of red blood cells; (**B**) M-MPDA@CeO_2_ Results of blood WBC(**a**), RBC(**b**), PLT(**c**), HGB(**d**), ALT(**e**), AST(**f**), BUN(**g**) and Cr(**h**) after 2 weeks of injection in mice compared with control group; (**C**) H&E staining analysis of major experimental tissues(heart, liver, spleen, lung, and kidney) of Balb/c mice after 2 weeks injection of M-MPDA@CeO_2_ compared with control group(Scale: 200 μm, *n* = 4); (**D**) Weight change curves of Balb/c mice(Control and M-MPDA@CeO_2_ groups) receiving different treatments on their 0,2,4,6,8,10,12 and 14 days(*n* = 4)

While numerous advantages of nanomaterials have been proposed, growing concerns have also been raised regarding their potential biosafety risks to human health. To investigate the hemolytic toxicity of the M-MPDA@CeO₂ nanocomposite, an in vitro hemolysis assay was conducted by co-incubating red blood cells with the nanomaterial, with ddH₂O served as the positive control and PBS as the negative control. As shown in Fig. 5A, even at a concentration of 300 µg/mL, the hemolysis rate of M-MPDA@CeO₂ was calculated to be only (2.09 ± 0.24) %. This result indicates that the M-MPDA@CeO₂ nanocomposite exhibits minimal interaction with blood, does not induce significant hemolysis, and demonstrates good blood compatibility, meeting the hemolysis test standards required for biomedical materials.

To evaluate the potential systemic toxicity of M-MPDA@CeO₂ during in vivo application, routine blood parameters, serum biochemical indicators, and body weight changes in mice were monitored after two weeks of continuous administration. The routine blood test results showed that all parameters detected by the hematology analyzer remained within the normal range after two weeks of M-MPDA@CeO₂ administration, with no significant differences compared to the control group (Fig. 5B). The serum biochemical indicators also revealed no notable differences between the M-MPDA@CeO₂ treatment group and the control group. Additionally, no significant abnormalities were observed in major organs such as the heart, liver, spleen, lungs, and kidneys of the treated mice compared to the control group (Fig. 5C). Body weight monitoring further indicated no marked differences between the M-MPDA@CeO₂-treated group and the control group throughout the administration period (Fig. 5D). These findings collectively suggest that M-MPDA@CeO₂, at the current dosage, exhibits no significant toxic or adverse effects and demonstrates favorable biocompatibility in vivo.

### Therapeutic efficacy of M-MPDA@CeO_2_ in ALI mouse model

**Fig. 6:**
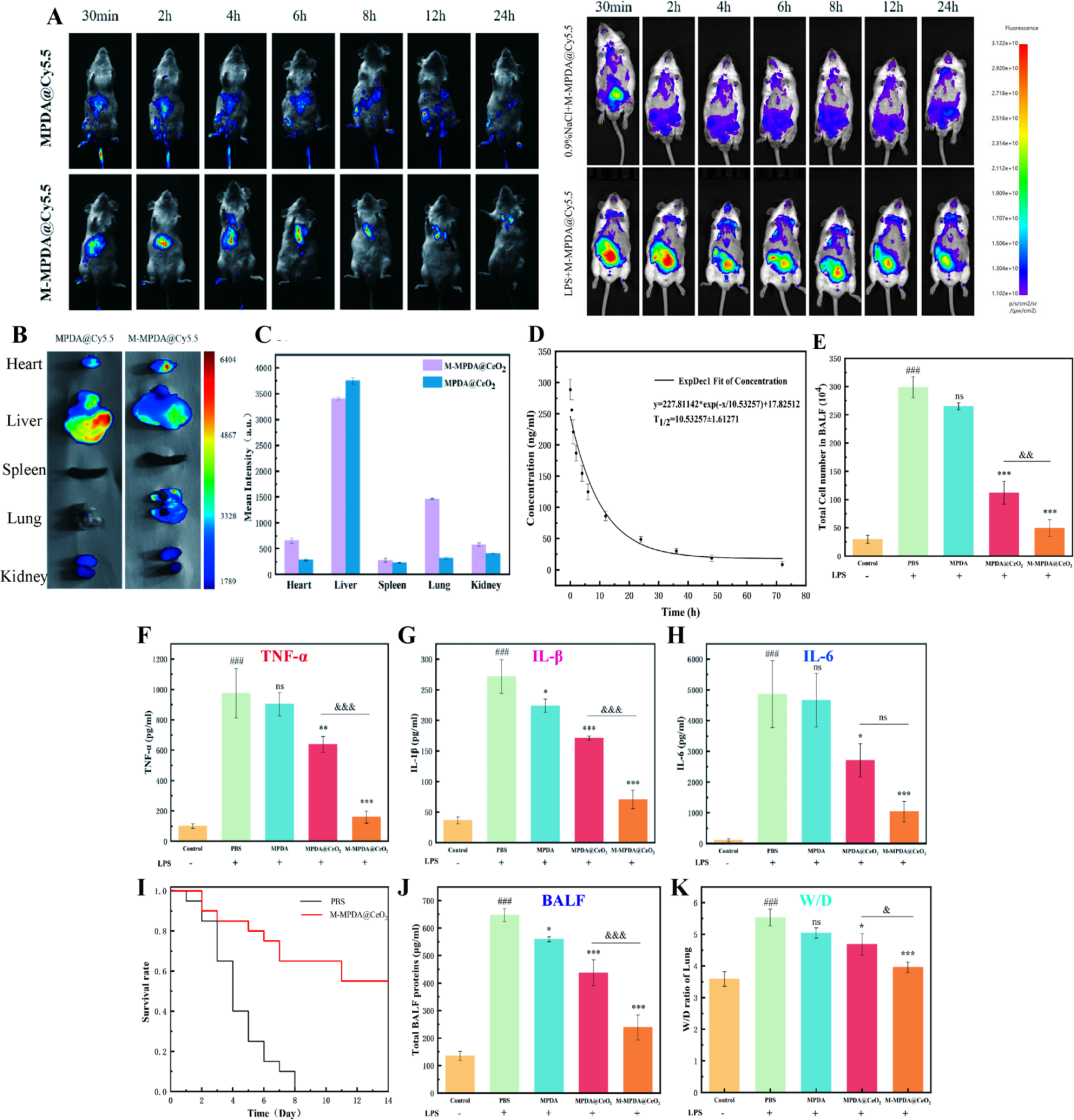
Biodistribution and Anti-inflammation Efficacy of M-MPDA@CeO_2_ in vivo. (**A**) In vivo fluorescence imaging of mice with different treatments and injection of MPDA@Cy5.5 or M-MPDA@Cy5.5 at different times; (**B**) Fluorescence images of the heart, liver, spleen, lung and kidney of ALI mice 24 h after caudal intravenous injection of MPDA@Cy5.5 or M-MPDA@Cy5.5 nanomaterials; (**C**) Quantification of fluorescence intensity in the heart, liver, spleen, lung and kidney of ALI mice; (**D**) Plasma concentration-time curve of Ce after intravenous injection of (**E**) Total number of cells in alveolar lavage fluid; (**F**-**H**) The contents of (**a**) TNF-α, (**b**) IL-1β and (**c**) IL-6 in alveolar lavage fluid of mice in different treatment groups were detected by Elisa. (**I**) M-MPDA@CeO_2_ survival curve of mice in the intervention group and the control group. (**J**) Total BALF protein concentration (µg/mL) of lung lobes. (**K**) Total protein concentration in alveolar lavage fluid. Wet/dry ratio of lung lobes

After administering the nanomaterial via tail vein injection into ALI mice, the fluorescence distribution was observed at different time points. As shown in Fig. 6A, relatively clear fluorescence signals began to appear in the lungs as early as 4 h after injection of M-MPDA@Cy5.5, with the signals subsequently becoming predominantly concentrated in the pulmonary region. In contrast, no significant fluorescence enrichment was observed in the lungs after injection of macrophage membrane-uncoated MPDA@Cy5.5. Compared to the non-injured cells treated with 0.9% NaCl, the M-MPDA@CeO_2_ nanocomposite uptake in LPS-treated cells is promoted (Fig. 6A), suggesting that nanocomposite uptake is specific to injured cells.

Mice were sacrificed 24 h post-injection, and major organs were harvested for fluorescence imaging (Fig. 6B and C)). In both groups, strong fluorescence signals were detected in the liver and kidney tissues, suggesting that the nanocomposite is primarily metabolized through the hepatic and renal pathways. Compared to the MPDA@Cy5.5 group, the M-MPDA@Cy5.5 group exhibited stronger fluorescence intensity in lung tissue, indicating that the macrophage membrane-coated nanocomposite can effectively accumulate at sites of inflammatory lung injury. This experiment further elucidates the dynamic process of in vivo distribution and accumulation of the nanocomposite and verifies that macrophage membrane-based biomimetic nanomaterials can mimic the surface characteristics and biological activities of macrophages. Through such biomimetic nanomaterials, precise targeting of lung injury sites can be achieved.

To elucidate the circulatory pattern of M-MPDA@CeO₂ in the blood, the concentration of cerium in mouse blood at different time points was quantitatively analyzed using ICP-OES after nanomaterial injection. As shown in Fig. 6D, the blood half-life of M-MPDA@CeO₂ was 10.53 ± 1.61 h. This prolonged circulation time is likely attributable to the macrophage membrane coating, which effectively helps the nanoparticles evade rapid phagocytosis by macrophages. Such an evasion mechanism contributes to maintaining a longer blood half-life for M-MPDA@CeO₂ in vivo, thereby enhancing its stability and pharmacokinetic performance.

The total cell count in BALF is commonly used to assess the degree of pulmonary inflammation, as inflammatory responses lead to the migration of leukocytes (particularly macrophages and neutrophils) from the bloodstream into the alveoli, thereby increasing the total cell count in BALF. A cell counter was used to determine the total cell count in the BALF of mice from each group. The cell count in the LPS model group was significantly higher than that in the control group (*P* < 0.001), reaching (298.89 ± 18.6) × 10⁴, confirming the successful establishment of the ALI mouse model (Fig. 6E). Compared to the model group, the total BALF cell count in the treatment groups decreased to varying degrees. Specifically, the LPS+MPDA@CeO₂ and LPS + M-MPDA@CeO₂ treatment groups showed reductions in total BALF cell counts to (111.77 ± 20.19) × 10⁴ and (49.43 ± 14.95) × 10⁴, respectively.

The concentrations of TNF-α, IL-1β, and IL-6 in the BALF supernatant were also measured. As shown in Fig. 6F-H, compared to the control group, the LPS-induced model group exhibited a substantial increase in the levels of pro-inflammatory cytokines TNF-α, IL-1β, and IL-6, indicating an uncontrolled inflammatory response in vivo. After intervention with different treatment groups, all groups demonstrated varying degrees of reduction in inflammatory cytokine levels. Notably, the M-MPDA@CeO₂ group showed a significant decrease in the concentrations of inflammatory cytokines, suggesting that this treatment strategy exhibits excellent efficacy in markedly suppressing inflammatory responses. An ALI mouse model was established using a lethal dose of LPS (50 mg/kg), and the survival times of mice in the M-MPDA@CeO₂ intervention group and the control group were recorded. By day 8, all 20 mice in the control group had died, while by day 14, more than half of the mice in the M-MPDA@CeO₂ intervention group remained alive (Fig. 6I).

In lung injury assessment, the protein concentration in BALF is regarded as a key indicator for evaluating changes in pulmonary capillary permeability. An increase in protein concentration in BALF directly reflects the extent of lung injury and the state of capillary permeability. As shown in Fig. 6J, compared to the control group, the LPS model group exhibited a significant increase in total protein concentration, reaching (647.61 ± 23.26) µg/mL, indicating enhanced alveolar epithelial microvascular permeability and substantial protein extravasation following LPS stimulation. After intervention with the nanomaterials, the BALF protein concentrations in the treatment groups decreased to varying degrees. Specifically, the LPS+MPDA@CeO₂ and LPS + M-MPDA@CeO₂ treatment groups showed reductions in BALF protein concentrations to (437.27 ± 46.55) µg/mL and (238.53 ± 45.34) µg/mL, respectively.

The wet-to-dry weight (W/D) ratio of the lung serves as a direct method for measuring the degree of pulmonary edema and is also a highly sensitive indicator for evaluating lung tissue damage. An increase in edema typically reflects abnormal accumulation of fluid in lung tissue, which is closely associated with the severity of lung injury. Twenty-four hours after modeling, the wet-to-dry weight of the right lung was measured in mice from each treatment group. The results are presented in Fig. 6K. Compared to the control group, the wet-to-dry weight ratio in the model group showed a significant increase (*P* < 0.001), indicating marked pulmonary edema. When compared to the model group, the MPDA treatment group did not exhibit a statistically significant change in the wet-to-dry weight ratio, while the MPDA@CeO₂ treatment group showed a slight decrease (*P* < 0.05). Most notably, the M-MPDA@CeO₂ treatment group demonstrated a significant reduction in the wet-to-dry weight ratio, suggesting that M-MPDA@CeO₂ exhibited the best efficacy in suppressing pulmonary edema.

### Determination on tregs cell proportion an*d* tissues oxidative stress injury in ALI

**Fig. 7:**
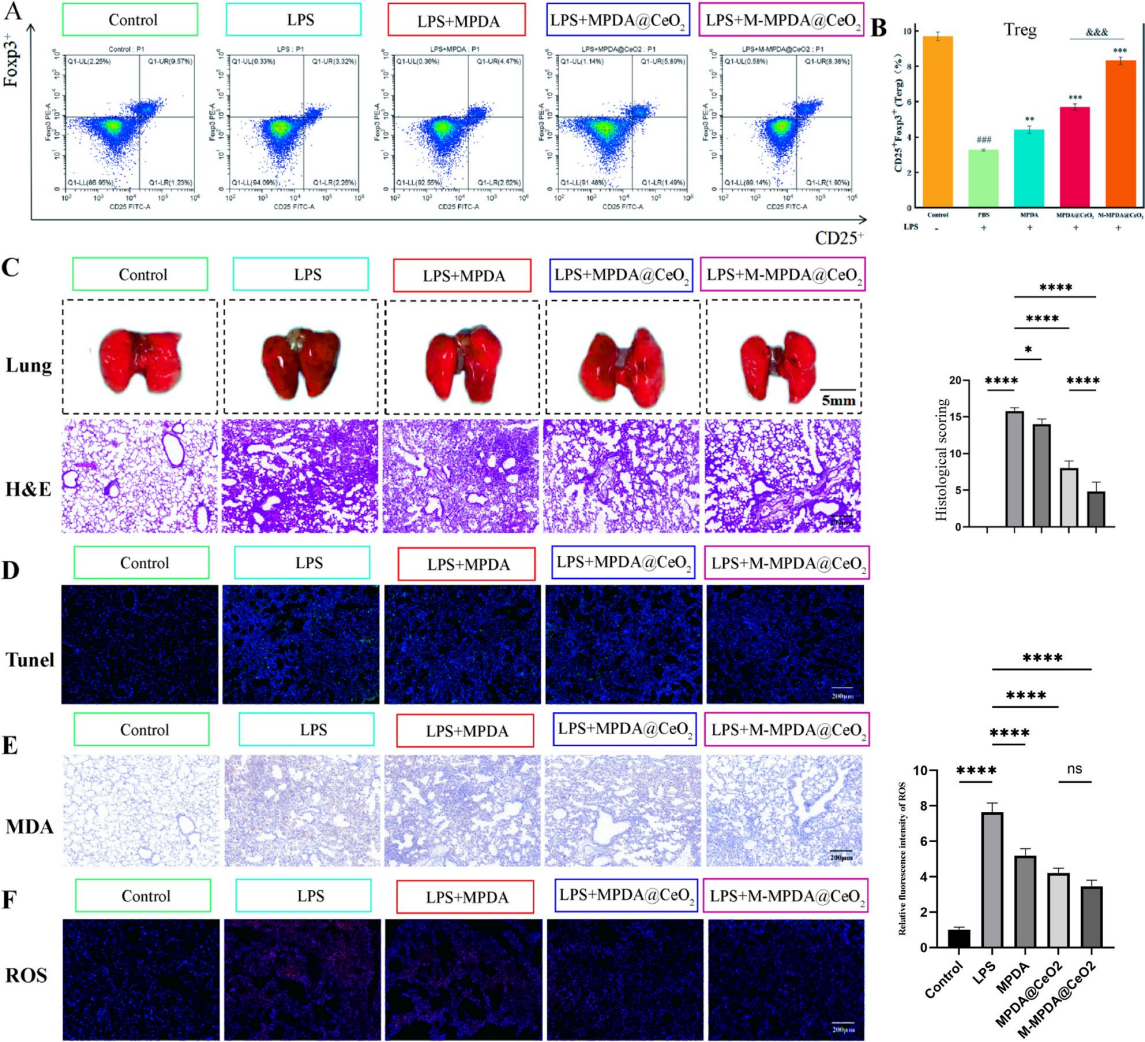
Antioxidative stress efficacy of M-MPDA@CeO_2_ in vivo. (**A**) The proportion of Tregs cells in peripheral blood of mice in different treatment groups was detected by flow cytometry; (**B**) Quantitative proportion of Tregs cells in peripheral blood of mice in each treatment group; (**C**) General view of lung tissue and H&E staining of lung lobes in each treatment group (Scale: 200 μm); (**D**) Tunel staining of lung tissue in each treatment group (Scale: 200 μm); (**E**) MDA immunohistochemical staining of lung tissue of mice in each treatment; (**F**) Reactive oxygen species staining of lung tissue in each treatment group (Scale: 200 μm) (*n* = 3,###*P* < 0.001 vs. control group; **P < 0.01 vs. model group; ***P < 0.001vs model group)

In the ALI mouse model, the proportion of peripheral blood Tregs [(3.27 ± 0.06) %] was significantly lower compared to the normal group [(9.68 ± 0.25) %] (*P* < 0.001) (Fig. 7A). Statistically significant differences were observed between the MPDA group [(4.41 ± 0.22) %], MPDA@CeO_2_ group [(5.69 ± 0.18) %], and M-MPDA@CeO_2_ group [(8.31 ± 0.2) %] and the model group (*P* < 0.05). After treatment with M-MPDA@CeO_2_, the proportion of peripheral blood Tregs significantly increased (Fig. 7B). Figure 7C demonstrated the H&E staining results: the LPS-induced model group exhibited extensive inflammatory cell infiltration in lung tissues, destruction of alveolar structures, and significant red blood cell leakage, leading to widened alveolar wall spaces. After treatment with different groups, the MPDA and MPDA@CeO_2_ groups showed varying degrees of improvement. The M-MPDA@CeO_2_ treatment group demonstrated the most pronounced improvement, significantly reducing alveolar septal thickness, alleviating inflammatory cell infiltration, and effectively suppressing interstitial and alveolar edema. TUNEL staining revealed scattered green fluorescence signals in the lung tissues of the LPS-induced model group, indicating tissue cell apoptosis following LPS stimulation (Fig. 7D). In the treatment groups, the green fluorescence signals in lung tissues gradually decreased, effectively demonstrating that the M-MPDA@CeO_2_ nanocomposite can protect lung tissues from oxidative stress damage.

MDA is one of the key byproducts of lipid peroxidation triggered by reactive oxygen species. Lipid peroxidation results from intracellular oxidative stress, and measuring MDA levels provides insight into the extent of lipid peroxidation, thereby assessing the degree of oxidative stress. The MDA content in lung tissues of mice from each group was detected via immunohistochemistry. The MDA measurement results are shown in Fig. 7E. In the LPS-induced model group, MDA levels significantly increased due to severe inflammation and lipid peroxidation. After various therapeutic interventions, a decreasing trend in MDA content was observed. Notably, treatment with M-MPDA@CeO₂ showed the most significant reduction in both inflammation and lipid peroxidation levels in vivo. This indicates that M-MPDA@CeO₂ possesses excellent potential in mitigating oxidative stress and inflammatory responses.

The results of ROS staining on frozen sections are shown in Fig. 7F. After LPS stimulation, lung tissues in the model group exhibited extensive red fluorescence across the entire field of view, indicating excessive accumulation of reactive oxygen species in the lung tissue due to ALI. This finding aligns with the previous experimental results. Following treatment with MPDA and MPDA@CeO₂, both materials demonstrated synergistic scavenging capabilities toward reactive oxygen species. After treatment with M-MPDA@CeO₂, reactive oxygen species in the injured lung tissues were significantly reduced. This further confirms that M-MPDA@CeO₂ exhibits outstanding reactive oxygen species scavenging ability in an in vivo environment. In a cecal ligation and puncture (CLP)-induced ALI model, we demonstrated that M-MPDA@CeO₂ exhibited similar pathological remission and ROS scavenging effects (Figure S2), proving reproducibility of the therapeutic effects in different ALI models.

## Discussion

Through interdisciplinary research combining advanced materials science and biomedical studies, this study proposes and elaborates on the design and investigation of an innovative nanocomposite material, which is consists of mesoporous polydopamine-loaded ultrasmall cerium dioxide nanoparticles and is modified with macrophage membranes (M-MPDA@CeO2). The study first focuses on the synthesis process of the M-MPDA@CeO2 nanocomposite, detailing aspects such as material formation, structure, and dimensions. Morphological analysis confirmed the successful coating of macrophage membranes on the surface of MPDA@CeO2, forming a core-shell structure. Western blot experiments verified the successful encapsulation of macrophage membranes, with the presence of key proteins detected. By precisely controlling the synthesis parameters, M-MPDA@CeO2 nanocomposites with excellent physicochemical properties were successfully prepared, including good dispersibility, stability, and superior antioxidant performance. Subsequently, a comprehensive evaluation of the M-MPDA@CeO2 nanocomposite was conducted, confirming its physicochemical characteristics, biocompatibility, and antioxidant capabilities in both in vitro and in vivo environments.

Macrophages are a type of immune cells with numerous proteins on their cell membrane surface, such as receptors for TNF-α, TRL2, and TRL4 [21]. These components endow macrophages with the ability to target bacteria and neutralize inflammatory factors. Macrophage membrane-coated nanoparticles have demonstrated high targeted delivery efficiency to various inflammatory diseases as well as decent therapeutic efficacy, including osteoarthritis [22], rheumatoid arthritis [23], cancer [24], microbial infections [25], and sepsis [26]. In this study, the cytotoxicity assay demonstrated that M-MPDA@CeO2 exhibited no significant toxicity at therapeutic concentrations. Moreover, due to the inheritance of the inflammatory recognition ability of natural macrophages, this biomimetic similarity enables the immune system to struggle in identifying these nanomaterials as foreign entities, thereby reducing the risk of clearance by the immune system [27]. It results in a longer circulation half-life in the bloodstream and an increased retention time within the body, thereby increasing the therapeutic effect. In a published report, M2 macrophage membrane-camouflaged Fe₃O₄-Cy7 nanoparticles have been proven to exhibit reduced immunogenicity in blood circulation, thereby enabling targeted NIR/MR imaging of atherosclerosis [19]. Consequently, M-MPDA@CeO2 nanocomposite coated by macrophage membrane enables high efficacy, low toxicity, increased biocompatibility, and reduced immunogenicity in treating ALI through a triple mechanism of biomimetic targeting, ROS scavenging, and macrophage polarization regulation.

Under LPS stimulation, excessive ROS-induced oxidative stress damage leads to loss of mitochondrial membrane potential, mitochondrial condensation, and degradation of the extracellular matrix, ultimately resulting in cell apoptosis [28]. Scavenging ROS plays a crucial role in mitigating oxidative stress damage and suppressing inflammatory responses [29]. CeO₂ has garnered significant attention due to its antioxidant properties, as it can scavenge ROS and act as an antioxidant [30]. This endows cerium with broad application potential in the field of biomedicine, particularly in antioxidant therapy. There are oxygen vacancies on the surface of cerium oxide nanoparticles (CeONPs), which can continuously scavenge ROS through reversible Ce³⁺/Ce⁴⁺ conversion [31], and inhibit ROS-induced mitochondrial damage, DNA breakage, cell apoptosis, and it blocks the NF-κB and MAPK pathways activated by ROS, reducing the release of pro-inflammatory factors [32]. CeONPs also inhibits M1 phenotype markers (iNOS, CD86) [33]. In this study, intracellular ROS detection results indicated that M-MPDA@CeO2 effectively reduces intracellular ROS levels, thereby alleviating inflammatory responses and protecting cells from oxidative stress damage. Additionally, our data revealed that M-MPDA@CeO2 suppresses the expression of M1 macrophage markers (iNOS, CD86) and promotes the expression of M2 macrophage markers (Arg1, CD206). These findings collectively indicate that M-MPDA@CeO_2_ possesses the potential to regulate the polarization of M1 macrophages toward M2 macrophages, effectively inhibiting LPS-induced inflammatory responses.

The biosafety of nanomaterials has long been a major concern. Yin et al. developed a class of PDA coated CeO_2_ nanozyme, and evaluated its biosafety in LPS-induced ALI rat model [34]. It demonstrated that this nanocomposite presented outstanding in vivo biosafety, not affecting the body weight, blood indicators and pathological features, exhibiting promising prospects in clinical application. In this study, we assessed the biocompatibility and safety of the M-MPDA@CeO_2_ nanocomposite in an LPS-induced ALI mouse model. By evaluating the hemolytic activity, physiological and biochemical indicators, body weight changes, and pathological alterations in major organs, we demonstrated the high biocompatibility and safety of M-MPDA@CeO_2_ at therapeutic doses. In the above LPS-induced ALI rat model, the PDA coated CeO_2_ nanozyme was proven with potent capability in decreasing lung inflammation, alleviating diffuse alveolar damage, as well as promoting lung tissue repair [34]. In this study, the targeting capability of the nanocomposite to lung inflammatory injury sites was validated by injecting M-MPDA@Cy5.5 via the tail vein and tracking its distribution in mice using small animal in vivo imaging technology. The results showed that fluorescence signals significantly accumulated in the lungs, indicating the excellent lung-targeting ability of the nanocomposite. Subsequently, we assessed the therapeutic effect of M-MPDA@CeO_2_ in ALI mouse model, and found that M-MPDA@CeO_2_ significantly alleviated LPS-induced lung injury, reduced the expression of inflammatory cytokines, and cleared ROS. In summary, this study not only validated the safety of M-MPDA@CeO2 in ALI mouse model and confirmed its outstanding anti-inflammatory and antioxidant properties in vivo. Only male BALB/c mice were used in this study to avoid potential confounding effects of the estrous cycle on inflammatory responses and oxidative stress, which could introduce variability in evaluating the therapeutic efficacy of M-MPDA@CeO₂. Previous studies on ALI have also predominantly used male mice to establish reproducible disease models [35]. Nevertheless, we acknowledge that sex differences may influence treatment outcomes, and future studies incorporating female mice are warranted to validate the translational potential of this nanocomposite in both sexes. Another limitation of this study is the absence of direct lung function measurements, such as oxygen saturation, dynamic compliance, or airway resistance, which are clinically relevant endpoints that directly reflect respiratory physiology. While our histological and biochemical data (e.g., ROS levels, inflammatory cytokine profiles, and wet/dry ratio) demonstrated the protective effects of M-MPDA@CeO₂ against oxidative stress and inflammation in ALI, these endpoints do not fully capture real-time pulmonary function. Future studies incorporating non-invasive pulse oximetry or invasive plethysmography are warranted to comprehensively evaluate the translational potential of this nanocomposite. Additionally, survival studies would further strengthen the clinical relevance of our findings.

This study successfully constructed M-MPDA@CeO_2_, a macrophage membrane-biomimetic nanocomposite consisting of mesoporous polydopamine loaded with cerium dioxide, and systematically investigated its protective effects against oxidative stress damage in ALI. Compared with PEGylation or other coatings, our macrophage membrane-coated M-MPDA@CeO2 offers distinct advantages, including excellent biocompatibility, immune evasion, and lesion-targeting capability, achieving precise drug delivery and release, thereby enhancing therapeutic efficacy. MPDA is a biocompatible nano-photosensitizer with high drug-loading efficiency; it exhibits efficient cellular uptake and superior intracellular ROS scavenging activity without inducing cytotoxicity. This study elucidated its key biological effects in regulating inflammatory responses and scavenging ROS. Furthermore, in vivo experiments validated the safety and efficacy of M-MPDA@CeO_2_ in an ALI mouse model. For clinical translation, our M-MPDA@CeO_2_ nanocomposite is intended to alleviate inflammatory responses and ROS-mediated oxidative stress injury in the case of sepsis-associated ARDS, with the feasibility of delivery route and loading dose exactly explored.

In conclusion, the lung-targeted drug delivery by M-MPDA@CeO_2_ provides new insights for treating pulmonary diseases, highlighting the broad prospects of nanotechnology in medical applications. Future research will further optimize the drug-loading capacity of M-MPDA@CeO_2_, aiming to offer more effective and safer nanomedical solutions for the treatment of pulmonary diseases. In the in vivo environment, nanozymes may be affected by protein adsorption from biological fluids, enzyme-mediated degradation, and clearance by the immune system, which can impact their long-term stability and bioactivity. Therefore, in vitro experiments and in vivo animal studies are required to evaluate the distribution, metabolism, and activity changes of nanozymes at different time points and in various tissues. Additionally, further in-depth research is needed on their interactions with biological tissues, metabolic pathways, and in vivo excretion to understand their behavior and long-term stability within the body.

## Supplementary Information

Below is the link to the electronic supplementary material.

Figure S1. Flow cytometry analysis of macrophage CD86 and CD206 expression in bone marrow derived macrophages treated with M-MPDA@CeO_2_

Figure S2. Antioxidative stress efficacy of M-MPDA@CeO2 in ALI model induced by CLP. (A) General view of lung tissue in each treatment group; (B) H&E staining of lung lobes in each treatment group (Scale: 200 μm); Reactive oxygen species staining of lung tissue in each treatment group (Scale: 200 μm) (n=3)

## Data Availability

The data in the present study are available upon reasonable request from the corresponding author.
